# From gene to dose: Long-read sequencing and *-allele tools to refine phenotype predictions of *CYP2C19*

**DOI:** 10.3389/fphar.2023.1076574

**Published:** 2023-03-01

**Authors:** Lonneke J. Graansma, Qinglian Zhai, Loes Busscher, Roberta Menafra, Redmar R. van den Berg, Susan L. Kloet, Maaike van der Lee

**Affiliations:** ^1^ Department of Clinical Pharmacy and Toxicology, Leiden University Medical Center, Leiden, Netherlands; ^2^ Leiden Genome Technology Center, Department of Human Genetics, Leiden University Medical Center, Leiden, Netherlands

**Keywords:** long-read sequencing, CYP2C19, personalized medicine, pharmacogenomics, bioinformatics

## Abstract

**Background:** Inter-individual differences in drug response based on genetic variations can lead to drug toxicity and treatment inefficacy. A large part of this variability is caused by genetic variants in pharmacogenes. Unfortunately, the Single Nucleotide Variant arrays currently used in clinical pharmacogenomic (PGx) testing are unable to detect all genetic variability in these genes. Long-read sequencing, on the other hand, has been shown to be able to resolve complex (pharmaco) genes. In this study we aimed to assess the value of long-read sequencing for research and clinical PGx focusing on the important and highly polymorphic *CYP2C19* gene.

**Methods and Results:** With a capture-based long-read sequencing panel we were able to characterize the entire region and assign variants to their allele of origin (phasing), resulting in the identification of 813 unique variants in 37 samples. To assess the clinical utility of this data we have compared the performance of three different *-allele tools (Aldy, PharmCat and PharmaKU) which are specifically designed to assign haplotypes to pharmacogenes based on all input variants.

**Conclusion:** We conclude that long-read sequencing can improve our ability to characterize the *CYP2C19* locus, help to identify novel haplotypes and that *-allele tools are a useful asset in phenotype prediction. Ultimately, this approach could help to better predict an individual’s drug response and improve therapy outcomes. However, the added value in clinical PGx might currently be limited.

## 1 Introduction

One drug does not have the same effect for everyone; inter-individual differences in drug response can lead to toxicity and drug inefficacy (Weinshilboum and Wang, 2004). A large part of this variability is caused by genetic variants in genes, called ‘pharmacogenes’, which are involved in the pharmacokinetic and pharmacodynamic processes occurring as part of drug metabolism (Scharfe et al., 2017; Tafazoli et al., 2021). Variants in these pharmacogenes are associated with diverse drug responses. After the discovery that over 97% of the general population carries at least one pharmacogenomic (PGx) variant which can potentially affect drug response (Dunnenberger et al., 2015), personalized medicine is slowly becoming standard of care instead of a ‘one size fits all’ approach. This implementation of PGx in clinical practice to guide treatment decisions is crucial to maximize the effectiveness of a treatment and minimize harm.

**FIGURE 1:**
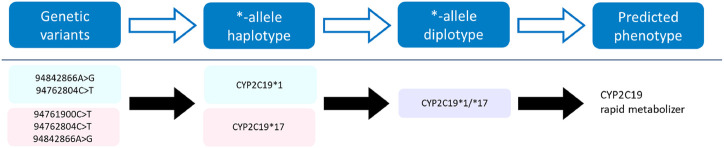
Visualization of the process towards predicting a phenotype. Genetic variants of the *CYP2C19* gene on the maternal (*pink*) and paternal (*blue*) allele are assigned a haplotype, which combined form a diplotype (*purple*). This diplotype is translated into a predicted phenotype on which the treatment will be based.

The pharmacogene *CYP2C19* is a member of the cytochrome P450 (CYP) superfamily and is involved in the metabolism of many commonly prescribed drugs such as clopidogrel and proton-pump-inhibitors (harmGKB). Moreover, *CYP2C19* is highly polymorphic; 50%–65% of the population is characterized with an not ‘normal’ metabolic capacity based on the genetic make-up of their *CYP2C19* resulting in the need for dose adjustments (Hicks et al., 2017). This combination of clinical relevance and high abundance of genetic variants makes it a highly important pharmacogene which is frequently tested in hospital laboratories to guide treatment decisions (Pratt et al., 2018). In order to use genetic information of *CYP2C19*, the star (*)-allele nomenclature and accompanying predicted phenotypes are used (Figure 1). Based on the combination of genetic variants identified, a haplotype or “*-allele” is assigned to the maternal and paternal allele according to the *-nomenclature system (Ingelman-Sundberg et al., 2000; Gaedigk et al., 2018). The two *-haplotypes are then combined into a diplotype. Based on the enzyme activity corresponding to the assigned diplotype, this diplotype is translated into a predicted phenotype (Figure 1). Different predicted phenotype categories are recognized which are used in the dosing guidelines of the Clinical Pharmacogenetics Implementation Consortium (CPIC) (Caudle et al., 2017) and the Dutch Pharmacogenetics Working Group (DPWG) (Swen et al., 2008). For *CYP2C19,* CPIC defines five different metabolizer types: Poor Metabolizers (PM), Intermediate Metabolizers (IM), Normal Metabolizers (NM), Rapid Metabolizers (RM) and Ultra-rapid Metabolizers (UM). DPWG on the other hand does not use the RM predicted phenotype. To aid in the interpretation of the high amount of variants that can be detected with sequencing approaches, *-allele tools have been developed. There is a great variety in these bioinformatic tools; each tool is based on a different reference database and genome, runs on different software and supports different input file formats (Table 1)*.* Since they all make different assumptions, the output of each tool may differ. A detailed analysis of the methods behind each tool, the performance of each tool and an analysis of their applicability using long-read sequencing is yet to be performed.

**TABLE 1:** Overview of *-allele tools and their specifications. For every *-allele tool selected, the supported input file format(s), the used reference genome, the prerequisite software and the reference database on which the *-allele tool analysis is based are depicted. When ‘WGS’ is stated, these tools assume input files with WGS data. ‘Tweak’ indicates that an adjustment was necessary before the *-allele tool supported this input file.

		Stargazer	Aldy^a^	PharmCat^a^	Cyrius	StellarPGX	PharmaKU^a^	PharmVIP^a^
Input format	VCF	X	X (tweak)	X			X	X
	BAM	X	X		WGS	WGS		X
	CRAM		X		WGS	WGS		
	SAM		X					
Reference Genome	GRCh37	X	X		X	X	X	
	GRCh38		X	X	X	X	X	X
Prerequisite Software	Windows	X						
	Linux	X	X			X		
	MacOS	X	X			X		
	Java			X				
	DRAGEN				X			
	Webbased						X	X
Reference database	PharmVar	X	X			X		
	PharmGKB		X	X			X	(PharmCat)^b^
	Own					X		
	Other		X				X (CPIC)^b^	

^a^ *-allele tools assessed in this study.

b This is based on the tool/database specified.

BAM, binary alignment map; CRAM, Compressed Reference-oriented Alignment Map; SAM, sequence alignment map; VCF, variant call format; WGS, whole genome sequence.

In the *-allele nomenclature, 71 *CYP2C19* variants are currently taken into account, of which only three are recommended for standard clinical testing by the association for molecular pathology (Pratt et al., 2021). In routine PGx for both clinical practice and research purposes, the variants of interest are generally genotyped with SNV (Single Nucleotide Variant) arrays (Mukerjee et al., 2018). These arrays directly interrogate the genomic positions of known variants. However, in Gnomad a total of 975 variants have been reported in *CYP2C19* so far. The fact that routine PGx uses a limited number of variants means that they are unable to identify all variants (including rare and novel variants). Moreover, the variants that are detected cannot be phased to their allele of origin. Phasing determines whether variants are located on the same allele or on opposing alleles (e.g., *CYP2C19* *2/*3 (PM) or *CYP2C19* *1/*2+*3 (IM)) which can be of major importance for enzyme activity. Hence, phasing is expected to improve haplotype assignment and therefore phenotype prediction (van der Lee et al., 2022). These two limitations, (novel) variant detecting and haplotype phasing, are hindering the ability of SNV arrays to fully characterize pharmacogenes.

To resolve these limitations, improve phenotype prediction and get a better understanding of genetic make-up, more advanced technologies are needed. While Next-Generation short read Sequencing (NGS) can help to identify all variants within the genes of interest (van der Lee et al., 2020a), it remains difficult to resolve complex regions and to perform direct (read based) haplotype phasing with short reads (100–200 bp). However, long-read sequencing has the ability to overcome the limitations mentioned above, since it is able to resolve larger regions and enables us to look into phased haplotypes. Due to the length of the reads (∼5.000 base pairs (bp) for capture based approaches), variants can be assigned to their allele of origin (Midha et al., 2019). While single pass accuracy is still limited with Pacific Bioscience (PacBio, 2023) HiFi sequencing, the circular consensus reads - which are obtained by combining multiple sequencing passes of the same molecule—are highly accurate (Wenger et al., 2019). To date, long-read sequencing for PGx has only been applied in a single gene setting (Qiao et al., 2016; Borràs et al., 2017; van der Lee, 2021) or with publicly available data of one individual (van der Lee et al., 2022). Recently, a long-read sequencing panel using PacBio technology, which consists of a selection of clinically relevant pharmacogenes, has been developed at the Leiden University Medical Center (LUMC).

While the application of long-read sequencing for clinical PGx might still be too costly and time intensive, due to the rapid decline in costs it can be expected that long-read sequencing originating from research applications becomes more abundantly available. This offers the opportunity to repurpose this data to extract a PGx profile using all *-allele variants and the phasing information. Hence, it is of importance to assess the performance of long-read sequencing for clinical PGx as well as for research. Therefore the aim of this study is to investigate the benefit of long-read sequencing for both of these applications, by resolving the *CYP2C19* locus, identifying (novel) variants, and assessing the performance of different *-allele tools. The knowledge gained from this exploratory study might give a focus and direction for further PGx research*.*

## 2 Methods

### 2.1 Long-read sequencing panel

48 samples originating from old PGx studies performed at the clinical pharmacy and toxicology department of the Leiden University Medical Center (LUMC), were available and anonymized. These samples were sequenced using the PacBio long-read sequencing panel developed by the department of clinical pharmacy and toxicology and the department of human genetics of the LUMC. This panel includes core PGx genes as well as genes that were of interest for specific projects at the departments. For every gene, ∼10.000 bp upstream and downstream of the transcription start and end site, respectively, were included in the panel. In short, DNA concentration and quality were checked by using the Qubit Fluorometer and the Qubit dsDNA Broad Range Assay kit (Invitrogen, Carlsbad, CA) and verified on the Femto Pulse system (Agilent Technologies, CA, United States). Subsequently, the DNA was sheared to an average size of ∼8 kb using the Diagenode Megaruptor three and purified using washed AMPure XP beads (Beckman-Coulter Woerden, Netherlands). End repair, A-tailing and adapter ligation was performed using 500 ng of sheared DNA product and the Twist Library Preparation Kit 1 (Twist Bioscience, CA, United States ) and the PacBio annealed barcoded adapter (10 μM, desalt-purified (Integrated DNA Technologies (IDT), Coralville, IA)). After further purification and size selection (3.7x diluted washed AMPure XP beads), the DNA was amplified using the Takara LA Taq HotStart kit (TaKaRa Bio United States, Inc.). The reaction was performed in two reaction volumes (100 μL) containing: 50–100 ng of DNA, 0.5 μM PacBio universal primer (/5Phos/gcagtcgaacatgtagctgactcaggtcac (IDT, Coralville, IA)), 0.1 mM of each dNTP, 1x LA PCR buffer, and 0.03 U Takara LA Taq. The PCR parameters were 2 min at 95°C, followed by six cycles of 20 s at 95°C, 15 s at 64°C and 10 min at 68°C, and a final extension of 5 min at 68°C. After amplification the two reaction volumes were pooled and the product was checked for concentration and quality with the Qubit Fluorometer and the Femto Pulse system. The product of eight samples was equimolarly pooled. Next, the capture was performed using the Twist Hybridization and Wash Kit (Twist Bioscience, CA, United States) and the Twist Probe Custom panels (Twist Bioscience, CA, United States). The pools were then amplified using the Takara LA Taq HotStart kit in two reaction volumes (100 μL) each containing: 50 μL captured sample pool, 0.5 μM PacBio universal primer, 0.2 mM of each dNTP, 1x LA PCR buffer, and 0.03 U Takara LA Taq. After another step of quality control with the Qubit Fluorometer and the Femto Pulse system, sequence libraries were prepared using 500 ng of the captured pooled samples. The library was sequenced on the Sequel^®^ II (Pacific Biosciences, CA, United States) on a 8 M SMRT cell at an on-plate concentration of 80 p.m. with the following specifications: sequencing primer V4, Sequencing kit 2.0 and binding kit 2.0 and a 30 h movie time. HiFi CCS (Circular concensus reads) were obtained for further processing (Wenger et al., 2019; PacBio, 2023).

### 2.2 Data preprocessing

The data was preprocessed using the LUMC developed variant calling pipeline specifically for the PacBio PGx sequencing project (Redmar van den Berg, 2021). The CCS subreads were demultiplexed using LIMA (Lima, 2023). Duplicate reads were marked using pbmarkdup (Pdmarkdup, 2023). Next, demultiplexed CCS bam files were mapped to the reference genome (GRCh38) using pbmm2 (Pbmm2, 2023). Thereafter, the variant calling was performed with GATK4 (GATK, 2023) and phased using WhatsHap (Martin et al., 2016). Finally, the results were aggregated and reported with MultiQC (Multiqc, 2023). The output was reported in both BAM (Binary Alignment Map) and VCF (Variant Call Format) files for each sample. Samples with less than 10% of the target bases reaching at least 30X coverage were excluded from the analysis.

All variants will be described according to Human Genome Variation Society (HGVS) nomenclature for GRCh38 (den Dunnen et al., 2016), using genomic positions on chromosome 10 NC_000010.11 (location of *CYP2C19*). If more applicable, dbSNP Reference SNP (RefSNP or rs) numbers are used (Sherry et al., 2001), as they are widely known and recognized in the PGx field.

### 2.3 Variant characterization

For variant characterizations, all VCF files were cross-referenced with a bed file containing the genomic coordinates of the start and end positions of the included genes to obtain a VCF file specific to the PGx genes. Moreover, clinically relevant variants were flagged based on their presence in PharmVar (PharmVar, 2022) or, if the gene was not available in PharmVar, their presence in the Ubiquitous pharmacogenomics consortium’s (U-PGx) variant panel (upgx, 2020; van der Wouden et al., 2019). Thereafter, the number of known variants (variants present in Pharmvar and/or the U-PGx panel) and novel variants (variants not used in the clinic) identified per gene were calculated using Excel. In this study, we refer to variants which are not in current PGx nomenclature as novel variants. For *CYP2C19* all clinically relevant variants were obtained from PharmVar.

The predicted impact of *CYP2C19* variants was assessed using the Ensembl Variant Effect Predictor (VEP) (McLaren et al., 2016) including SIFT (Kumar et al., 2009) and PolyPhen (Adzhubei et al., 2010) for missense variants and the Combined Annotation Dependent Depletion (CADD) score (Rentzsch et al., 2021) was used to predict the magnitude of the impact of non-synonymous variants, selecting on *CYP2C19* (transcript: ENST00000371321.9). The ∼10.000 bp upstream and downstream regions were also taken into account during the impact analysis. While *in silico* algorithms have a limited accuracy in predicting enzymatic function (80% accuracy) (Han et al., 2017), we use them here as one of the tools to explore the potential impact of (novel) variants as no PGx specific *in silico* tools are available. The CADD score was selected as prediction tool to assess what part of the identified variants is classified as high impact (CADD score >10) and is a candidate for further functional assessment. It has previously been shown that, of the non-class specific tools, the CADD score performs best on pharmacogenes (Han et al., 2017).

### 2.4 *-Allele calling and phenotype prediction

For every sample the predicted metabolizer type was assigned based on the identified genetic variants in *CYP2C19*. To investigate the performance of *-allele tools on long-read sequencing data and to study potential differences in underlying assumptions of these tools, a comparison was made between manual *-allele assignments and assignments from four different *-allele tools. Manual curation was used as the ground truth as we could check phasing and variants manually for all samples, thereby omitting the risk of inaccurate phasing assumptions. Moreover, the results were used to determine the predicted phenotypes of the samples according to the CPIC guidelines (Caudle et al., 2017). To ensure a broad selection, these four tools were selected based on three criteria: 1) every tool uses reference genome GRCh38, 2) all tools support VCF as input file and 3) they all require different software to run. For the automatic processing, four *-allele tools were selected: PharmVIP (Piriyapongsa et al., 2021), Aldy v3.0 (Numanagić et al., 2018), PharmaKU (John et al., 2021) and PharmCat 1.8.0 (Sangkuhl et al., 2020). PharmVIP and PharmaKU were used as described in the literature (Piriyapongsa et al., 2021) (John et al., 2021). Since they are both web-based, no installations were necessary. In order to run Aldy, Ubuntu 20.04.4 was installed inside VirtualBox (6.1), Aldy was run using the VCF option by changing the default from hg19 to hg38. PharmCat was run on Java 16.0.2. Since these tools use the CPIC definition of the metabolizer types, the CPIC guidelines were also used for the manual assignment. The manual assignment was performed using the phased VCF files and the PharmVar database of *CYP2C19* (June 2022) (PharmVar, 2022).

Due to limitations in the processing of samples in the PharmVIP tool, a complete analysis of all samples could unfortunately not be performed. PharmVIP limits the storage of the results to 10 days and ten samples at any one time, making it not applicable for clinical practice and not suitable for this study. Hence, the remaining three *-allele tools are assessed in more detail and no PharmVIP analyses are included in the results.

The results of the tool-based analyses were combined into one file, after which the manual *-allele assignments were added. Based on this, the accuracy (compared to the manual curation) and ease of use of the different *-allele tools and added value of phasing were assessed.

### 2.5 Statistical analysis

If not specifically stated, analyses were performed using the software as described in the tool documentation. Data was processed with Excel, R version 1.4.1717 and Python 3.9.12. Visualizations were performed with R version 1.4.1717 and Adobe Illustrator 25.2.3.

## 3 Results

### 3.1 Variant identification

To assess the ability of long-read sequencing to detect (novel) genetic variants, the number of variants identified per gene was analyzed. Out of the 48 samples, 11 samples are excluded from the study due to a lack of coverage (<10% Target Bases 30X) (Supplementary Figure S1). For the remaining 37 samples, an average of 84.1% of the target bases reached 30X coverage (range: 33%–96%). Moreover, the average read length was 5,418 bp (range: 3,980–6,277 bp) and the average haploblock size 7,507 bp (range 4,863–9,806 bp). After this exclusion, 813 unique *CYP2C19* variants can be identified in the entire *CYP2C19* region (including upstream and downstream regions) of which 13 are present in the current PGx nomenclature. 303 of the 813 variants are singletons; they are only detected once in our cohort. The same trend is observed for the other genes in the panel, where many more variants are identified compared to the number currently used in the guidelines (Supplementary Table S1). This discrepancy remains when only looking at the core gene (without taking the flanking regions into account). For *CYP2C19,* 683 variants are identified in the core gene (start to end position of the gene) of which 8 variants are present in PharmVar. Besides identifying the *CYP2C19* variants in the cohort, the variants were also phased. In total, an average of 76% (11%–100%) of the variants in the *CYP2C19* locus could be phased to their allele of origin in relation to at least one other variant.

**FIGURE 2:**
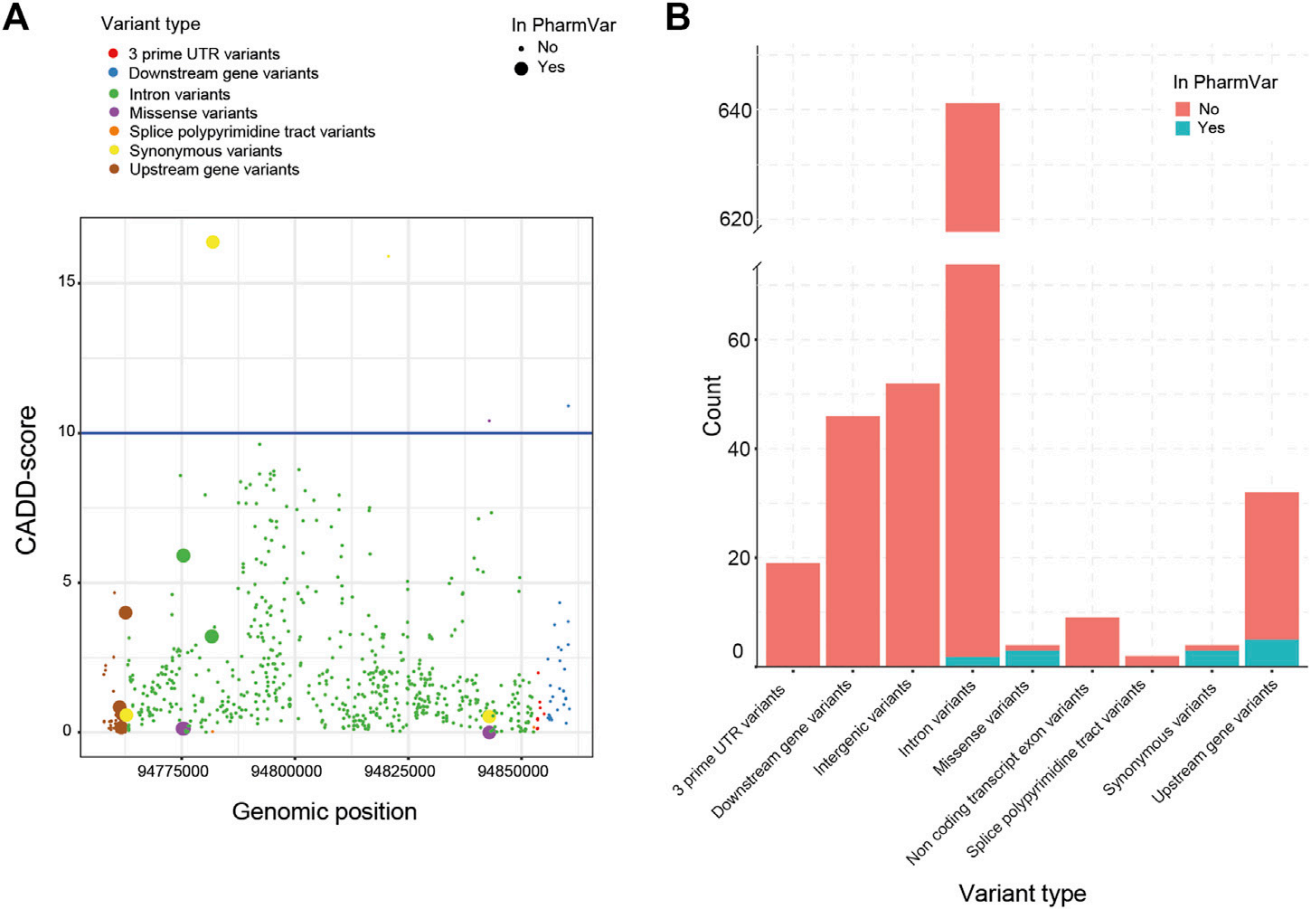
Variant impact analysis for identified *CYP2C19* variants. The variant impact analysis by VEP, resulted in Combined Annotation Dependent Depletion (CADD) scores (*A, left*) and predicted variant effect (*B, right*), for every identified variant of *CYP2C19* in our cohort. **(A)** Available CADD-scores of all detected *CYP2C19* variants. The blue line marks the cut-off CADD score of 10. **(B)** Predicted variant effect of all detected *CYP2C19* variants. Clinical variants are based on the PharmVar database.

Next, we investigated the predicted impact of all 813 variants using the Combined Annotation Dependent Depletion (CADD)-score generated by the Ensembl Variant Effect Predictor (VEP). Based on these CADD-scores, no relationship between the presence of a variant in the current nomenclature and its CADD-score could be established (Figure 2A). This lack of association between CADD-scores was visible for all VEP categories (e.g., missense, splice site, upstream). In total, only four variants had a CADD score above the cut-off of 10. The most common clinical deleterious variant in our cohort (g.94781859G>A, *2) was assigned the highest score (16.4). The other three variants are not part of *-allele nomenclature (one synonymous, one missense and one downstream).

All variants found in the cohort that are present in PharmVar are either upstream (*n* = 5), intronic (*n* = 2), synonymous (*n* = 3) or missense variants (*n* = 3) (Figure 2B). For the other variants identified in the cohort, these numbers were: upstream (*n* = 27), intronic (*n* = 643), synonymous (*n* = 1) and missense variants (*n* = 1), the remainder of the variant types is shown in Figure 2B. This enrichment of variants in the missense category in the clinical group (3 missense variants of 13 known variants) compared to the novel group (1 missense variant out of 675 novel variants) can be expected as the non-coding variants are often not regarded as impactful and may not be included in the guidelines and nomenclature as rapidly as variants in coding regions are. The four missense variants were predicted to be not deleterious by SIFT (tolerated) and PolyPhen (benign). Three of these missense variants are part of the *-alleles (PharmVar, 2022). The first (g.94775507G>A>) is characteristic for *11 which is associated with a normal function allele. The second (g.94842866A>G) is a core variant of multiple *-alleles including *1 and is not expected to have an impact on enzyme function. The third missense variant (g.94775165G>C) is not a core variant and only associated with three minoralleles of *2 (*2.002, *2.010 and *2.012).

Out of the four missense variants identified in the entire cohort, three are in the PharmVar database. The fourth missense variant is not present in PharmVar even though literature confirmed the potential deleterious effect of this variant (g.94842860C>T) (Medicine Nlo. rs59734894, 2022; PharmGKB, 2022) and the CADD score is 10.4. The variant is reported to be associated with a decreased expression of *CYP2C19* compared to the reference allele, which would lead to a decreased metabolic capacity of CYP2C19 and subsequent dose adjustment. The individual carrying this variant was characterized as a *1/*17, unfortunately due to a decrease in coverage in intron five for this individual we are unable to determine if the novel variant (exon 7) is on the same allele as the *17 variant (upstream) or not.

### 3.2 Phenotype prediction

The data of the identified *CYP2C19* variants was used for a manual and *-allele tool based *-allele prediction.

#### 3.2.1 Manual

**FIGURE 3:**
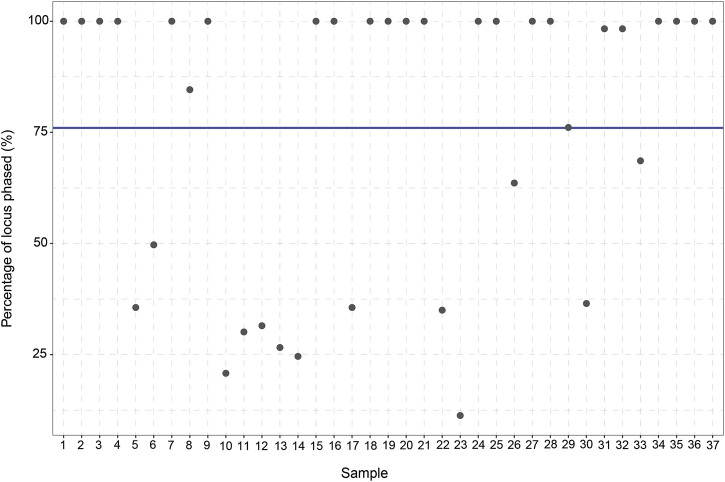
Percentage of *CYP2C19* variants phased to their allele of origin per sample. For every sample (*n* = 37) the percentage (%) of the variants which could be phased to their allele of origin was calculated. The blue line displays the average percentage of phased variants for the 37 samples (76%).

The manual *-allele assignment resulted in an overview of the haplotype predictions for every sample*.* The *1/*17 (RM) diplotype was observed to be the most frequent diplotype in our cohort (35%) and only eight samples (21.6%) did not have any actionable PGx variants. Six of the 37 patients (16.2%) were carriers of CYP2C19*38.003 + *g*.94781616A>G which is not yet part of the *-allele nomenclature. All six samples showed 100% phasing (Figure 3), affirming that g.94781616A>G is indeed present on the CYP2C19*38 allele. According to PharmVar, the intronic g.94781616A>G variant is currently only associated with CYP2C19*3.002 and not present in any other (minor)*-alleles. This makes it a potential novel (minor)*-allele with a relatively high frequency.

#### 3.2.2 *-Allele tools

Based on the criteria put (GRCh38 as reference, support of VCF files and different platforms as their basis), three *-allele tools were selected; Aldy, PharmaKU and PharmCat. Advantages and disadvantages of these tools are described in Supplementary Table S2.

Assignments from Aldy were in agreement with the manually assigned diplotypes based on major (e.g., *2) and minor alleles (e.g., *2.002) for 56.8% of the assignments (*n* = 21) (Table 2). Due to this tool’s transparency, we could observe that the Aldy resource file was not up to date with PharmVar as several *-alleles were not included, explaining the differences between the manual prediction and the assessment of Aldy. Moreover, Aldy is the only tool which also returns minor alleles (PharmaKU and PharmCat only return major alleles). When only looking at the major alleles, as they are most commonly used in clinical practice, the prediction by Aldy overlapped for 81.1% with our manual assignment (*n* = 30). Unfortunately, the Aldy output does not include predicted phenotypes or drug guidelines.

**TABLE 2:** Overview of haplotype prediction by different *-allele tools. The 37 samples were assessed by different *-allele tools (Aldy, PharmaKU and PharmCat) and manually. The predicted haplotypes are depicted for each sample.

Samples	Manual, *-alleles (predicted phenotype)	Aldy, *-alleles (predicted phenotype)	PharmaKU, *-alleles (predicted phenotype)	PharmCAT^b^
1	*1.002/^a^38.003 + 94781616A>G (NM)	*1.001 + rs17885098/*1.005 (NM)	*1/*1 (NM)	*1/*5
2	*1.002/*38.003 + 94781616A>G 6 (NM)	*1.001 + rs17885098/*1.005 (NM)	*1/*1 (NM)	*1/*5
3	*1.002/*17.001 (RM)	*1.002/*17.001 (RM)	*1/*17 (RM)	*1/*4 (*1/*17)^a^
4	*1.002/^b^17.001 (RM)	*1.002/*17.001 (RM)	*1/*17 (RM)	*1/*4 (*1/*17)^a^
5	*1.002 +94842865C>T/*2.002–94842865C>T (IM)	*1.002/*2.002 (IM)	*1/*2 (IM)	*1/*2
6	*1.002/*1.002 (NM)	*1.002/*1.002 (NM)	*1/*1 (NM)	*1/*1
7	*1.002/*17.001 (RM)	*1.002/*17.001 (RM)	*1/*17 (RM)	*1/*4 (*1/*17)^a^
8	*1.002/^b^17.001 (RM)	*1.002/*17.001 (RM)	*1/*17 (RM)	*1/*4 (*1/*17)^a^
9	*1.002/^b^17.001 (RM)	*1.002/*17.001 (RM)	*1/*17 (RM)	*1/*4 (*1/*17)^a^
10	*1.002^b^*1.002 (NM)	*1.002/*1.002 (NM)	*1/*1 (NM)	*1/*1
11	*1.002/*17.001 (RM)	*1.002/*17.001 (RM)	*1/*17 (RM)	*1/*4 (*1/*17)^a^
12	*1.002^b^*1.002 (NM)	*1.002/*1.002 (NM)	*1/*1 (NM)	*1/*1
13	*1.002/*1.002 (NM)	*1.002/*1.002 (NM)	*1/*1 (NM)	*1/*1
14	*1.002/*1.006 (NM)	*1.002/*1.006 (NM)	*1/*1 (NM)	*1/*1
15	*17.001/*38.003 + 94781616A>G (RM)	*1.001/*17.001 (RM)	*1/*17 (RM)	*4/*38 (*17/*38)^a^
16	*1.002^b^*2.011 + 94775507G>A (IM)	*2.001/*11.001 + rs4986894 (IM)	*1/*2 (IM)	*1/*2
17	*1.002/*2.012 (IM)	*1.002/*2.002 (IM)	*1/*2 (IM)	*1/*2
18	*1.002/*17.001 (RM)	*1.002/*17.001 (RM)	*1/*17 (RM)	*1/*4 (*1/*17)^a^
19	*1.002^b^*17.001 (RM)	*1.002/*17.001 (RM)	*1/*17 (RM)	*1/*4 (*1/*17)^a^
20	*1.002^b^*38.003 + 94781616A>G (NM)	*1.001 + rs17885098/*1.005 (NM)	*1/*1 (NM)	*1/*5
21	*1.002/*17.001 (RM)	*1.002/*17.001 (RM)	*1/*17 (RM)	*1/*4 (*1/*17)^a^
22	*1.002^b^*17.001 (RM)	*1.002/*17.001 (RM)	*1/*17 (RM)	*1/*4 (*1/*17)^a^
23	*1.002^b^*1.002 (NM)	*1.002/*1.002 (NM)	*1/*1 (NM)	*1/*1
24	*1.002/*17.001 (RM)	*1.002/*17.001 (RM)	*1/*17 (RM)	*1/*4 (*1/*17)^a^
25	*1.002^b^*2.011 (IM)	*1.002 + rs4986894/*2.001 (IM)	*1/*2 (IM)	*1/*2
26	*1.002/*17.001 (RM)	*1.002/*17.001 (RM)	*1/*17 (RM)	*1/*4 (*1/*17)^a^
27	*1.002^b^*2.011 (IM)	*1.002 + rs4986894/*2.001 (IM)	*1/*2 (IM)	*1/*2
28	*1.002/*17.001 (RM)	*1.002/*17.001 (RM)	*1/*17 (RM)	*1/*4 (*1/*17)^a^
29	*1.002^b^*1.002 (NM)	*1.002/*1.002 (NM)	*1/*1 (NM)	*1/*1
30	*1.002/*2.011 (IM)	*1.002 + rs4986894/*2.001 (IM)	*1/*2 (IM)	*1/*2
31	*1.002/*2.011 (IM)	*1.002 + rs4986894/*2.001 (IM)	*1/*2 (IM)	*1/*2
32	*1.002/*2.011 (IM)	*1.002 + rs4986894/*2.001 (IM)	*1/*2 (IM)	*1/*2
33	*1.002/*1.002 (NM)	*1.002/*1.002 (NM)	*1/*1 (NM)	*1/*1
34	*1.002/*2.011 (IM)	*1.002 + rs4986894/*2.001 (IM)	*1/*2 (IM)	*1/*2
35	*1.002/*38.003 + 94781616A>G (NM)	*1.001 + rs17885098/*1.005 (NM)	*1/*1 (NM)	*1/*5
36	*2.011/*17.001 (IM)	*2.001/*17.001 + rs4986894 (IM)	*2/*17 (IM)	*2/*4 (*2/*17)^a^
37	*1.002^b^*38.003 + 94781616A>G (NM)	*1.001 + rs17885098/*1.005 (NM)	*1/*1 (NM)	*1/*5

^a^The haploype predictions depicted are the first predictions in the output list. The haplotype prediction between brackets is added, when the prediction corresponding to the manual assessment, was present in the list but not as first output.

^b^No phenoypes are assigned for PharmCat due to ambiguity in haplotype assignments.

NM, normal metabolizer; IM, intermediate metabolizer; RM, rapid metabolizer.

PharmaKU does include phenotype predictions and guidelines and predicted 83.8% of the major diplotype calls accurately compared to the manual analysis (*n* = 31) (Table 2). The simple upload screen makes PharmaKU an easy and clear tool to use. Unfortunately, the program behind PharmaKU is a black box compared to the transparent script of Aldy as the tool is fully webbased. After uploading the VCF file of interest, the output file starts downloading automatically. Moreover, the analysis only includes major *-alleles; minor alleles are not taken into account.

The final *-allele tool tested was PharmCat. Since this tool reports all possible haplotypes for a sample, with only 34 variants as reference database, the prediction accuracy was rather poor compared to the manual prediction (45.9% overlap, *n* = 17) when selecting the first reported diplotype (Table 2). This is due to the software’s algorithm which assumes any genomic positions not present in the VCF file to be missing and not wildtype. With background knowledge or a prior manual assessment, the accurate prediction was frequently present in the output list (only not as the first *-allele assignment) increasing the accuracy to 86.5% compared to the manual assignment. Similar to PharmaKU, the output only includes core *-alleles, but it does include drug guidelines and hyperlinks to literature with more information.

The high degree of phasing in these samples was shown to be of importance in assigning the *CYP2C19* haplotypes for multiple samples (e.g., sample 25 and sample 27). These samples were manually assigned with a CYP2C19*1.002/*2.011 diplotype. However, when using *-allele tool Aldy, these samples were predicted to be CYP2C19*1.002 + rs4986894/*2.001 (Supplementary Figure S2). Rs4986894 corresponds to *g*.94762608T>C, which is the variant responsible for the distinction between CYP2C19*2.001 and CYP2C19*2.011; CYP2C19*2.001 + rs4986894 is CYP2C19*2.011. Due to the 100% phasing in those samples (Figure 3)*,* it can be concluded with certainty that the rs4986894 is present on the CYP2C19*2 allele. Hence, the correct haplotype would be CYP2C19*1.002/*2.011. Other tools (PharmCat and PharmaKU) both assigned *1/*2 as predicted haplotype to these samples, as these tools do not use minor alleles the discrepancy was not seen there).

Overall, 35% of the individuals were regarded as normal metabolizers based on the manual assignments. A further 27% was IM and 38% was classified as RM. No individuals were classified as PM or UM. PharmCat phenotypes were not assigned due to the ambiguity in the genotyping as described above. For the remaining tools there was no discrepancy on a phenotype level (Table 2).

## 4 Discussion

In current routine PGx, phenotype predictions are most commonly based on SNV array data. Despite the advantages of this method, such as rapid turnaround times and a straightforward interpretation, it also has limitations; it is not possible to detect all variants and direct (read based) phasing cannot be performed. In the present study, we assessed the performance of long-read sequencing and *-allele tools for the characterization of *CYP2C19*. We showed that with long-read sequencing, novel PGx variants can be identified and that the majority (on average 76% for the *CYP2C19* locus) of these variants can be phased to their allele of origin. Moreover, we have been able to identify a potentially impactful variant currently not used in PGx nomenclature as well as a novel minor allele of CYP2C19*38. Finally, the majority of the assessed *-allele tools result in accurate predicted phenotype assignments while the diplotypes did differ between the different tools. These findings show the benefits and potential of applying long-read sequencing in PGx for *CYP2C19* in a research setting and provide a glimpse into the future of clinical PGx.

Based on the long-read sequencing data, a missense variant (g.94842860C>T, *T* = 0.00013 in Caucasians (Medicine Nlo. rs59734894, 2022) in *CYP2C19* could be identified which, according to literature, decreases *CYP2C19* expression but is not (yet) included in the current PGx databases. Since the assigned diplotype of the patient carrying g.94842860C>T is *1/*17 (RM), which is associated with a higher activity of CYP2C19, carrying this missense mutation might have major consequences. It is possible that carrying this mutation reverses the higher enzyme activity due to CYP2C19*17, making this patient a normal or even intermediate metabolizer to whom different drug dosages apply than the recommended dose for a rapid metabolizer. Hence, this variant warrants further study and should, if previous findings are confirmed, be added to the PGx nomenclature. The same holds true for other variants identified. For example, splice site variants can have a high impact since they might result in aberrant proteins (Riolo et al., 2021) and a non-functional CYP2C19 enzyme. Intriguingly, none of the splice site variants detected in *CYP2C19* (*n* = 2) are present in the current PGx databases. One of these variants (g.94781806A>G) is located in intron four on the junction with exon five and the other (94852716A>G) in intron eight on the junction with exon 9 (Kent et al., 2002). No literature describing the potential impact of the variants could be found and they were assigned CADD scores below 10. Moreover, only a few specific intron variants are recorded in PharmVar. The emphasis lies on variants affecting the coding sequence, even though it has been proven that intronic variants can potentially create or disrupt a splice site, affecting the enzyme activity (Ingelman-Sundberg and Sim, 2010).

Using the CADD scores to identify potentially deleterious variants resulted in four variants with a CADD score of 10 or higher. One of these variants was a well-known clinical variant (the g.94781859G>A, *2), another was confirmed by literature to be potentially deleterious. The remaining two (one synonymous and one downstream) have an unknown impact. It should be kept in mind that many pharmacovariants have small effects (decreased function) which do not result in completely inactive or absent protein. These smaller effects do add up in a clinical setting but are difficult to predict with available *in silico* tools. Hence, high CADD scores might be useful to identify potential high impact variants but on the other hand a low CADD score does not mean that the variant has no impact on enzyme function.

Besides applying long-read sequencing for variant identification, it also enabled us to phase the identified variants back to their allele of origin using read backed phasing with WhatsHap. The variability in the phasing percentages can have two major causes. First, the read length might not have been sufficient due to DNA fragmentation, resulting in limited coverage. As we selected samples based on overall quality this is not likely to be the major cause. On the other hand, this fragmentation and lack of coverage can be the cause of the 11 samples that did not meet our criteria. The second reason for low phasing is a possible lack of heterozygous variants; to phase two reads together, forming a phasing haploblock, at least one heterozygous variants is needed.

Moreover, it is important to keep in mind that read backed phasing differs from statistical phasing, resulting in possible discrepancies between the *-allele assignments. This was seen in our study for multiple samples, where the manual assignment based on read backed phased VCF and BAM files was *CYP2C19* *1.002/*2.011 while *-allele tool Aldy, using statistical phasing, assigned *CYP2C19* *1.002 + rs4986894/*2.001. Conventionally, haplotypes are assigned using statistical phasing based on population statistics which might not be accurate for the individual. For those haplotype assignments, linkage disequilibrium (LD) is used. However, it is known that some variants can occur separately despite their strong linkage disequilibrium (van der Lee et al., 2020b) and the statistical phasing depends on the LD-threshold that is set (e.g., *r* ^2^ > 0.8 or *r* ^2^ > 0.85), which results in differences between the predictions. Read backed phasing, as with long-reads, is more accurate for individual patients.

Finally, the long-read sequencing data was used to predict the phenotype of every sample and to explore the performance of different *-allele tools. For six out of the 37 patients, the manual *-allele assignment identified a CYP2C19*38.003 haplotype. However, in all six cases, the clinical variant *g*.94781616A>G (rs7088784) was found on the same allele as CYP2C19*38.003, confirmed by closer analysis of the BAM files. This variant is currently only associated with CYP2C19*3.002 (PharmVar, 2022). In combination with the high frequency of this occurring in our cohort (16%), this might point towards a novel suballele of CYP2C19*38. This new minor allele would include all the variants of CYP2C19*38.003 plus g.94781616A>G, the shared variant with CYP2C19*3.002. It should be noted that g.94781616A>G is not a core SNV of CYP2C19*3, nor is it a variant with a high impact; according to PharmVar the variant impact is ‘No Change’. This would point towards a new minor allele without a clinical impact.

Interestingly, the *-allele assignment by *-allele tools did not show *g*.94781616A>G in their output at all. As it is only a minor mutation of CYP2C19*3.002, it is only reported if its parent allele is present. The absence of *g*.94781616A>G in the *-allele tool output illustrates how the *-allele tools work; they return the diplotype prediction in which most variants are accounted for and generally do not mention all additional variants observed, making the identification of possible new *-alleles by using *-allele tools challenging. Moreover, *-allele tools do not always agree as 45% of the calls in this study were the same in all tools based on the major alleles and the first diplotype presented by PharmCat. After manually checking the PharmCat diplotypes this agreement increased to 81%. This is largely caused by different assumptions made by the tools and the various (updates of) reference databases they use. For example, for one individual sample PharmCat predicted *17/*38, while *17/*1.001 was predicted by Aldy. This discrepancy can be explained by the fact that *38 is not included in the Aldy3 database. After adding *38 to Aldy’s database, all *38 alleles were detected and predicted accurately. On a phenotype level, there were no discrepancies between manual assignments, Aldy and PharmaKU indicating that the difference between the tools for our cohort were minor and not of clinical influence. However, care should still be taken as the difference could be of clinical relevance when they concern rare variants which are part of *-alleles nomenclature but maybe not yet present in all the tools algorithms. Furthermore, the most applicable tool will depend on the skills and interests of the user. For a physician, a straightforward tool with an accurate prediction and clear output would be ideal. Hence, physicians might favor PharmaKU. However, for the researcher with a grasp of bioinformatics and the urge to understand every detail, the more transparent and adaptable *-allele tool Aldy would be the first choice. Lastly, it is important to mention that most *-allele tools are dynamic tools and, as a result, are subject to change and updates.

While we were able to identify interesting variants and haplotypes, the sample size of this cohort is small. An analysis of more samples would yield more information and increase the power of our study. However, despite the small cohort of this study, we already identified novel variants and a novel minor allele. The fact that this is possible with only a limited number of samples strengthens our analysis; repeating this study for a larger cohort will presumably result in even more discoveries. Hence, it is anticipated that, by using long-read sequencing, new clinically relevant PGx variants and haplotypes will be discovered in the near future.

It is important to note the difference in requirements between clinical PGx and PGx in a research setting. In a clinical setting, quick results of well-known variants are needed to guide drug treatment. Although long-read sequencing is decreasing rapidly in costs and in turn-around time, it is not yet close to the SNP assays used in clinical practice. Therefore, the role of long-read sequencing in current PGx will be limited to those cases where conventional PGx cannot provide an answer or to cases where sequencing data is available and PGx can be extracted as a bonus. Nonetheless, as pre-emptive genotyping is slowly being adapted, the longer turn-around time of sequencing based PGx will become less of an issue and the added data that can be obtained will be a major benefit. Therefore, we envision that in the near future long-read sequencing based clinical PGx will become more common.

Non-etheless, one major limitation does remain: the impact of novel variants and haplotypes. While more and more variants are identified in sequencing based studies, the clinical impact of these variants is still unknown. Meanwhile, most clinical studies focus only on well-known and established variants with the use of SNP panels. The clinical data collected in these studies is of crucial importance to assign functional effects to novel variants and haplotypes. Efforts should be made to integrate these type of studies by adding sequencing to clinical PGx studies and clinical data to sequencing studies when possible. The same holds true for a clinical setting, when sequencing data is used for clinical PGx the outcomes of the patients treatment can be used to inform researchers and clinicians on potential effects of novel variants. Vice versa, sequencing data can help to identify novel and potentially deleterious variants which might cause an unexpected drug response.

The establishment of more cohorts with clinical data and advanced genetic data also offers the opportunity to develop better phenotype prediction tools. *-Allele tools offer the opportunity to assign haplotypes known within the current nomenclature. However, the current categorical system is unable to account for small individual variant effects and relies on classifying an individual into limited phenotype categories. Previous studies have shown that using more advanced phenotyping methods which predict drug response on a continuous scale substantially improves the explained variability of the drug metabolizing enzyme CYP2D6 (McInnes et al., 2020; van der Lee et al., 2021). For CYP2C19, similar models could be developed based on (long-read) sequencing data and clinical outcomes. However, datasets which have both advanced genetic data and clinical outcomes are limited.

In conclusion, our study highlights the value of long-read sequencing for PGx in regard to accurate phenotype prediction. It shines a light on the possible role that long-read sequencing can play, together with *-allele tools, in future clinical PGx and in research. This study only shows the tip of the iceberg and highlights that a new focus on computational tools and big data is required to ultimately improve our ability to predict drug metabolism and thereby drug outcomes for the individual patient.

## Data Availability

The datasets presented in this study can be found in online repositories. The names of the repository/repositories and accession number(s) can be found below: European Genome-Phenome Archive [https://ega-archive.org/], EGAS00001006929.
