# Supplementary material for: From gene to dose: Long-read sequencing and *-allele tools to refine phenotype predictions of CYP2C19
Source: Front Pharmacol. 2023 Mar 1;14:1076574. doi: 10.3389/fphar.2023.1076574 (PMC10014917; doi:10.3389/fphar.2023.1076574)
Supplement: Supplementary file 1 [file Table1.DOCX]

**Supplementary table 1 – Number of genetic variants identified by using long-read sequencing compared to number of known** **variants**For every gene, the number of variants found using long-read sequencing and the number of known variants (variants present in Pharmvar and/or the U-PGx panel) differs; more variants are identified in our cohort than the number of known variants. This trend is visible for the total region sequenced (gene including ~10.000bp up- and downstream), as well as for the core gene only (no flanking regions included).

| **Gene** | **Total** | | | | **Core** | | | |
| --- | --- | --- | --- | --- | --- | --- | --- | --- |
|  | Panel size (bp) | Variants found, n | Known variants, n | Known variants of variants found, n | Locus size (bp) | Variants found, n | Known variants, n | Known variants of variants found, n |
| *CYP2B6* | 36247 | 352 | 64 | 25 | 27116 | 266 | 51 | 17 |
| *CYP2C19* | 110524 | 813 | 71 | 13 | 92866 | 683 | 9 | 8 |
| *CYP2C9* | 70733 | 544 | 110 | 13 | 51433 | 369 | 91 | 6 |
| *CYP2D6* | 32964 | 569 | 358 | 54 | 4366 | 338 | 289 | 45 |
| *CYP3A4* | 165564 | 704 | 39 | 18 | 27288 | 393 | 36 | 10 |
| *F5* | 92422 | 826 | 1^a^ | 1 | 74637 | 726 | 1^a^ | 1 |
| *SLCO1B1* | 128044 | 1088 | 46 | 13 | 108602 | 983 | 44 | 11 |
| *TPMT* | 46763 | 481 | 3^a^ | 2 | 26766 | 227 | 3^a^ | 2 |
| *UGT1A1* | 33051 | 250 | 6^a^ | 0 | 13030 | 89 | 2^a^ | 0 |
| *VKORC1* | 24115 | 302 | 1^a^ | 1 | 5138 | 83 | 1^a^ | 1 |

^a^ Based on U-PGx data instead of PharmVar.
bp, base pair.

**Supplementary table 2 – Overview comparison *-allele tools**Every phenotype predictor has its advantages and disadvantages.

| **Phenotype predictor** | **Advantages** | **Disadvantages** | **Accuracy^a^** |
| --- | --- | --- | --- |
| Aldy | - Transparant - Adaptable | - Less user friendly^b^ - No drug guidelines | 81% |
| PharmaKU | - User friendly - Includes drug guidelines | - Not transparant - Not adaptable | 84% |
| PharmCat | - Medium transparency - Includes drug guidelines | - Only core *-alleles - Prior knowledge needed | 46% |
| Manual | - Transparant - Adaptable | - Time consuming - Error prone | Comparison group |

^a^ Only major alleles. Predictions without resource update (Aldy) or previous knowledge based on which the output is selected (PharmCat)
^b^  For users with no experience with bioinformatics

**
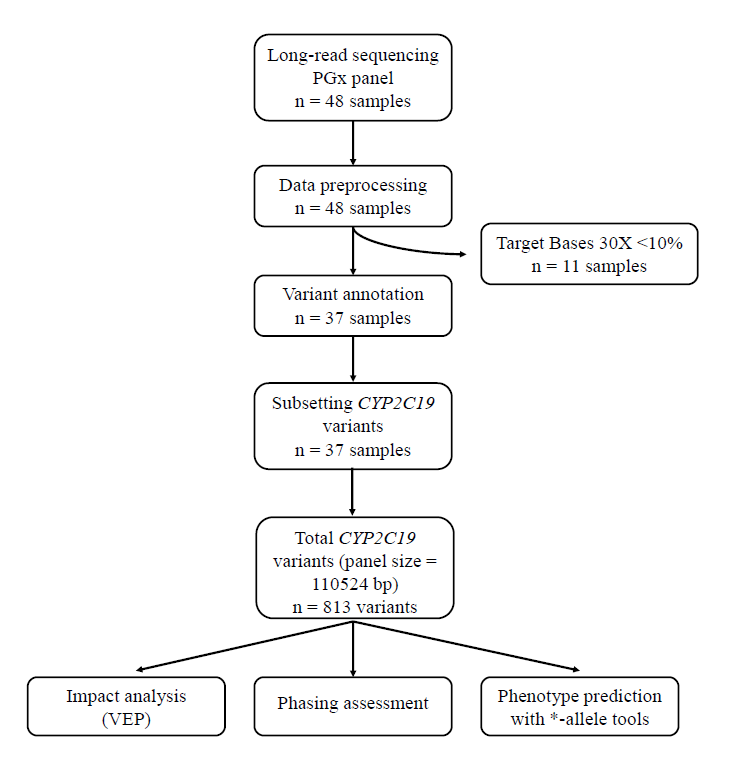
**

**Supplementary figure 1 – Schematic diagram of the analysis workflow**.


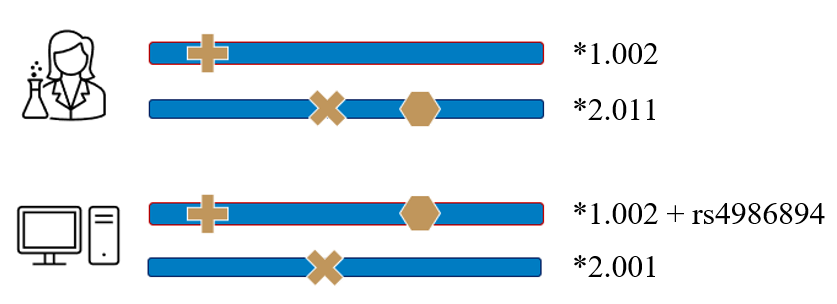


**Supplementary figure 2 – Comparison of read backed phasing *(upper)* and statistical phasing *(lower)* by Aldy.**

*CYP2C19**2.001 and *CYP2C19**2.011 which only differ in one variant (rs4986894) is shown as an example of the effect of different phasing assumptions. The manual *-allele assignment, *(upper)* resulted in *CYP2C19**1.002/*2.011, while the prediction based on *-allele tool Aldy *(lower)* returned *CYP2C19**1.002 + rs4986894/*2.001.
